# THE FORECASTING OF POPULATION SUPER-AGING AND POLICY SIMULATION OF LONG-TERM CARE IN CHINA (2019-2070)

**DOI:** 10.1093/geroni/igz038.915

**Published:** 2019-11-08

**Authors:** Chen Chen

**Affiliations:** Institute for Population and Development Studies, Zhejiang University, Hangzhou, Zhejiang, China

## Abstract

The purpose of this paper is to provide a policy simulation of Long-Term Care based on the aging population forecast for China, focusing on the super-aging and oldest-old segments of the population. As a developing country, it is a challenge for China to increase its wealth with the financial implications of an aging nation. The study identified two significant turning points for population development: 1) The multi-pilot program of long-term care policy reform must be executed between 2016 – 2020. This reform is time dependent because the multi-state longevity development period is from 2016 to 2030 and in the year 2030, the oldest-old population will be part of China’s society. 2) The year 2060 will be representative of a stable period in China, as the age of China’s population will no longer be accelerating.

